# Patient Perspectives of Quality Compared to Quantity of Life Regarding Orbital Exenteration

**DOI:** 10.1002/ohn.1364

**Published:** 2025-07-29

**Authors:** Kalpesh Hathi, Colin MacKay, Robyn Macfarlane, S. Mark Taylor

**Affiliations:** ^1^ Department of Surgery Division of Otolaryngology–Head and Neck Surgery, Dalhousie University Halifax Nova Scotia Canada; ^2^ Division of Medical Oncology Dalhousie University Halifax Nova Scotia Canada

**Keywords:** interview, length of life, oncology, orbital exenteration, quality of life, standard gamble, time trade‐off

## Abstract

**Objective:**

Orbital exenteration (OE) impacts patients cosmetically, functionally, and psychosocially. Eye‐sparing strategies with the advent of immunotherapy have developed the potential to avoid OE but may result in suboptimal oncologic outcomes and reduced survival. This study assesses patients' perceptions regarding quantity versus quality of life when considering OE compared to alternative treatment modalities.

**Study Design:**

Mixed‐methods study, utilizing quantitative health utility tasks and qualitative patient interviews.

**Setting:**

Tertiary care center.

**Methods:**

Fifty‐one patients previously treated for head or neck cutaneous malignancies completed interviews utilizing well‐established methodology to assess health state utility values (HSUVs) through time trade‐off and standard gamble tasks. This methodology assessed the level of risk patients would be willing to accept to avoid OE in the context of alternate treatment options. Open‐ended discussions regarding factors influencing decision‐making facilitated an inductive qualitative analysis highlighting patient priorities.

**Results:**

Patients were willing to accept 40.6% ± 28.7% risk of death or give up 3.2 ± 2.8 years of survival to avoid OE. This translated to an HSUV for OE of 0.68. The main factors influencing treatment decisions were (1) family, (2) healthcare perceptions, (3) age, (4) social consequences, and (5) risk tolerance.

**Conclusion:**

The consequences of OE on patients' quality of life impact their decision‐making. Patients may be willing to accept relatively high levels of risk to avoid OE. This highlights the importance of eye‐sparing strategies and shared decision‐making to ensure patient‐centered care, which may not be solely prioritized to survival when it comes to OE.

Advanced cutaneous malignancies can invade the orbit.^1^, ^2^, ^3^, ^4^ Malignant invasion of the orbit can necessitate orbital exenteration (OE).^1^, ^2^, ^3^, ^4^ OE involves resection of orbital contents and is often reconstructed using free‐tissue transfer.^1^, ^2^, ^3^, ^4^, ^5^, ^6^ The resulting cosmetic, functional, and psychosocial deficits from OE impact patients' quality of life.^7^, ^8^, ^9^, ^10^

In the past decade, immunotherapies for cutaneous malignancies have facilitated the development of “eye‐sparing” strategies to reduce morbidity with OE.^1^, ^9^, ^11^, ^12^, ^13^, ^14^, ^15^, ^16^, ^17^, ^18^, ^19^, ^20^, ^21^, ^22^, ^23^, ^24^, ^25^ Specifically, anti‐PD1 immunotherapy has shown a 50% complete pathologic response for resectable advanced cutaneous squamous cell carcinoma.^1^, ^11^, ^12^, ^13^, ^14^, ^15^, ^16^, ^17^ Dual checkpoint inhibition is the standard of care in advanced melanoma, and there is increasing evidence for neoadjuvant treatment of resectable disease.^1^, ^11^, ^18^, ^19^, ^20^ Similarly, basal cell carcinoma (BCC) has been addressed with anti‐SMO therapy.^1^, ^11^, ^21^, ^22^, ^23^, ^24^, ^25^ These advances could provide alternatives to OE, but oncologic and survival outcomes may be reduced. Understanding patient perspectives and preferences on OE in the context of eye‐sparing strategies is essential.

The trade‐off in quantity versus quality of life was first assessed in 1981 for total laryngectomy versus radiotherapy for laryngeal cancer.^26^ McNeil et al interviewed 37 healthy volunteers comparing radiotherapy, which provided lower survival rates but maintained functional voice, compared to total laryngectomy, which offered greater survival but the loss of normal speech.^26^ This study concluded that patients may forego survival benefits and choose radiation to maintain their quality of life (voice).^26^ Studies have built upon this topic, developing health utility methodology including time trade‐off (TTO) and standard gamble (SG).^27^, ^28^, ^29^, ^30^, ^31^, ^32^, ^33^, ^34^, ^35^, ^36^, ^37^, ^38^

This is the first study to assess the trade‐off in quantity versus quality of life for OE. This is increasingly important with growing evidence regarding immunotherapy, which may allow for eye preservation but suboptimal oncologic outcomes.

## Methods

### Study Design

Cross‐sectional videoconference patient interviews were performed. Approval from the Nova Scotia Health Authority Research Ethics Board was obtained (File # 1029834). Informed consent was completed using a standardized form on REDCap (Research Electronic Data Capture).^39^, ^40^, ^41^

### Patient Recruitment and Inclusion

All patients previously treated for a cutaneous malignancy or premalignant lesion of the head or neck by the study's senior author (S.M.T.) from April 1, 2020, to December 31, 2023, were approached for voluntary participation in the study. All interviews were performed by one study author (K.H.).

Videoconferencing was selected to avoid travel as a barrier to participation. The use of cameras and screen sharing provided both audio and visual cues. Patients treated for cutaneous malignancies or premalignant lesions of the head or neck were recruited as this was felt to be the most demographically representative of patients who may undergo OE.

Patients were excluded if actively undergoing oncologic treatments, unable to read and verbally communicate in English, lacked capacity, lacked access to videoconferencing, or had a previous or upcoming OE.

### Interview

A preset interview questionnaire/script was developed by the senior and primary study author (S.M.T. and K.H.) (Supplemental Appendix A, available online). Supplemental Appendix A, available online, was screen‐shared to facilitate visual cues. The interview began with demographic questions followed by two health utility assessments (TTO and SG), a health priority ranking task, and an open discussion. To ensure comprehension, patients were asked to explain their interpretation of each task before its commencement to the interviewer. Patients were encouraged to interject at any point for clarification.

### TTO Health Utility Assessments

TTO assesses the length of life in a suboptimal health state that individuals would be willing to forego to live in an improved health state.^35^, ^36^, ^37^ This study gave patients two hypothetical scenarios: (1) guaranteed 10 years of life after undergoing OE and (2) avoiding OE for reduced survival. Patients had the cosmetic and functional outcomes of OE explained to them in layman's terms and through visual cues. Patients were then asked to select living a guaranteed 10 years after undergoing OE or avoiding OE for a 9‐year lifespan. The lifespan when avoiding OE was decreased by 1 year at a time until the participant could no longer decide.

### SG Health Utility Assessments

SG assesses an individual's preferences in the presence of uncertainty.^36^, ^37^, ^38^ Patients were offered two hypothetical scenarios: (1) undergoing OE for a guaranteed 10 years of life and (2) taking a medication that avoids OE for an equivalent 10 years but confers with it a percentage risk of death. First, the participants were asked if they would rather have a guaranteed 10 years of life post‐OE or take the medication, which provides good health for 10 years but a 90% risk of death. The risk of death decreased by 10% until the participant could not decide.

### Health Priority Ranking and Open‐Ended Question

Participants were then asked to rank three factors in level of importance: (1) quality of life, (2) length of life, and (3) avoiding risks.

Finally, the interviewer asked patients what other factors played a role in their responses, and an open‐ended discussion proceeded. Patient responses were copied and coded as direct quotes.

Supplemental Appendix A, available online, facilitated delivery of these complex tasks; bar graphs visually contrasted the years of life and percent risk of death between the two options, images of patients post‐OE and full text of all questions were presented.

### Chart Review

Retrospective chart review collected treatment characteristics and patient demographics.

### Data Analysis

The cohort was described demographically. The results of the TTO, SG, and health priority ranking were reported descriptively. Continuous variables were reported as means ± standard deviation (SD) and categorical variables as percentages.

Fisher's exact tests assessed for uniformity amongst the health priority rankings. Pearson correlations assessed for a correlation between TTO and SG responses with age. Kruskal‐Wallis rank sum tests assessed for an association between TTO and SG responses with the type of malignancy. A priori alpha level of <.05 was deemed significant. Ordered logistic regressions were used to assess predictors of patient responses. Data analyses were performed using the R statistical packages.^42^

Health state utility values (HSUVs) were calculated using TTO results. The closer the HSUV is to 0.0, the greater value the patient places on avoiding the treatment outcomes.^35^, ^36^, ^37^, ^38^ The number of years the patient selected to live post‐OE was used to quantify the participants' HSUV. For example, if the patient selected to live 8 years in good health instead of 10 years post‐OE, but not 7 years, their HSUV regarding OE would be 7/10 = 0.7.^38^, ^39^, ^40^ Sensitivity analysis was performed to determine the impact on the mean HSUV by removing extreme values individually and together and comparing them to the base value.

The open‐ended responses underwent a thematic analysis using an inductive approach. A review of the text was completed, followed by coding responses into categories and larger themes, which were reported. Data were coded solely by the lead author (K.H.).

### Sample Size

A sample size of 50 was targeted. This sample size powered the 3 × 3 chi‐square (effect size [*w*]: 0.5; *α*: .05; power: 0.8; degrees of freedom [df]: 4; sample size: 48); and Pearson correlations (effect size|*P* |: .5; *α*: .05; power: 0.8; tails: 2; df: 24; sample size: 26). The primary objective was descriptive; however, a sample size of 50 is in line with the previous health utility and treatment‐decision‐making studies in head and neck oncology.^26^, ^29^, ^33^, ^43^, ^44^

Methodology described by Guest et al ensured qualitative data saturation. A base sample size of 15 interviews, a run length of five interviews, and a new information threshold of 0% was utilized.^45^

## Results

### Patient Inclusion

**Figure 1:**
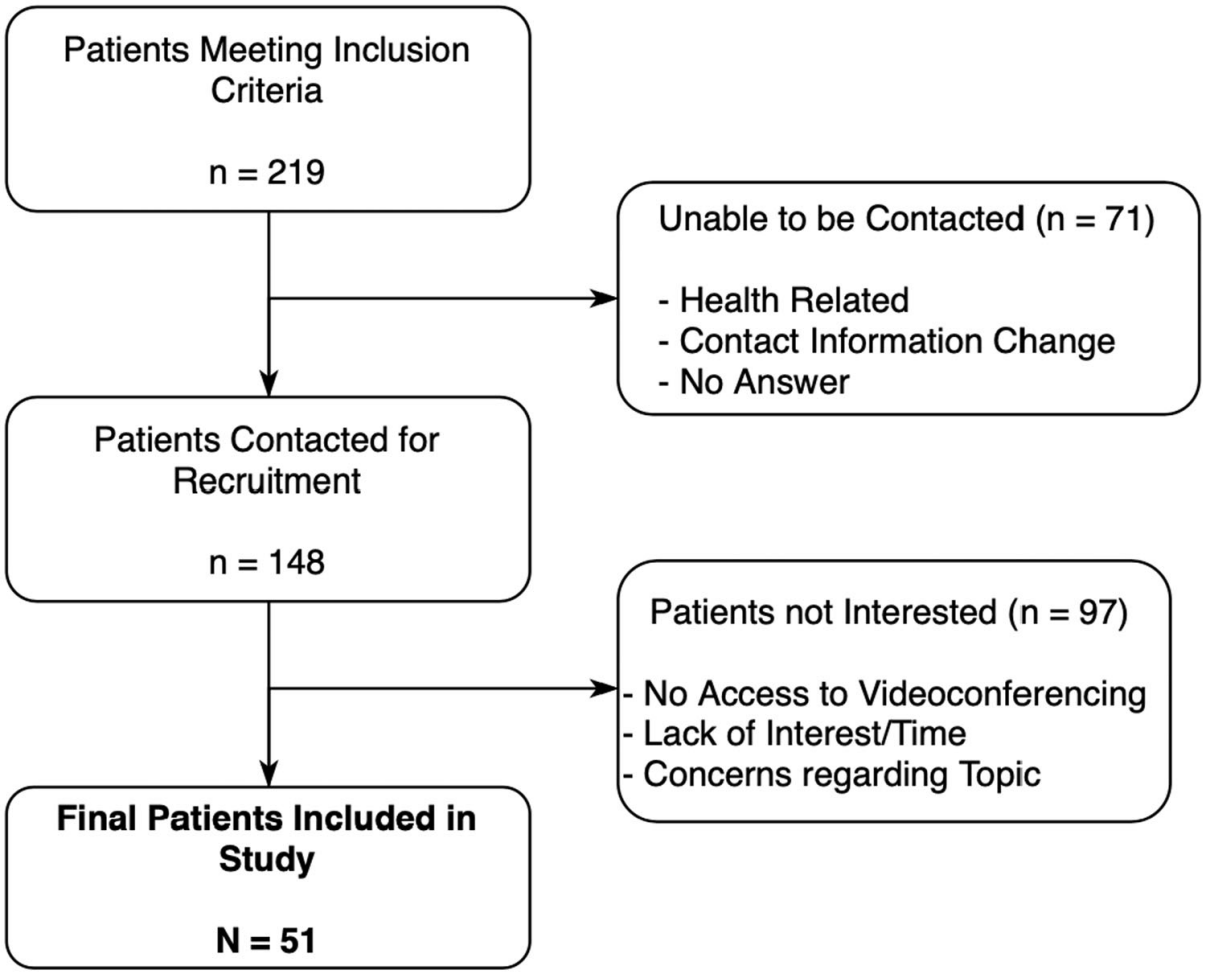
Patient inclusion and exclusion.

In total, 219 patients were eligible, and 71 were unable to be approached. Overall, 148 patients were approached, and 51 completed the interviews (Figure 1).

### Demographics

Table 1 reports patient demographics. The mean age was 68.6 ± 13.0 years, 56.9% (29/51) were male, 33.3% (17/51) had completed a bachelor's degree, and 41.2% (21/51) had an average household income of $50,000 to $99,999.

**Table 1:** Patient Demographics

Patient characteristic	N = 51 N (%)
Gender	
Male	29 (56.9)
Female	22 (43.1)
Highest level of education completed	
Did not complete high school	1 (2.0)
High school graduate	12 (23.5)
Community college	8 (15.7)
Bachelor's	17 (33.3)
Trade's education	2 (3.9)
Master's degree or higher	11 (21.6)
Average household income	
<$25,000	1 (2.0)
$25,000‐$49,999	3 (5.9)
$50,000‐$99,999	21 (41.2)
$100,000‐$149,999	10 (19.6)
$150,000‐$249,999	9 (17.6)
$250,000‐$500,000	4 (7.8)
>$500,000	3 (5.9)

BCC was the most common lesion treated, 24/51 (47.1%); the most common subsite was the ear, 14/51 (27.5%) (Table 2).

**Table 2:** Treatment of Patients Included in Study

Variable	N = 51 N (%)
Cutaneous lesion treated	
BCC	24 (47.1)
SCC	14 (27.5)
Melanoma	10 (19.6)
Actinic keratosis/cheilitis	3 (5.9)
Anatomic area impacted	
Ear	14 (27.5)
Nose	8 (15.7)
Cheek	8 (15.7)
Neck	5 (9.8)
Lips	5 (9.8)
Scalp	4 (7.8)
Eyebrow/lid	4 (7.8)
Forehead/temple	3 (5.9)
Surgical treatment	
Local anesthetic	37 (72.5)
General anesthetic	14 (27.5)
Surgical reconstruction	
Advancement/rotational flap	34 (66.6)
Pedicled flap	7 (13.7)
Primary closure	4 (7.8)
Full‐thickness skin graft	2 (3.9)
Regional or free flap	2 (3.9)
Shave/peel	2 (3.9)

Abbreviations: BCC, basal cell carcinoma; SCC, squamous cell carcinoma.

### TTO and SG

**Figure 2:**
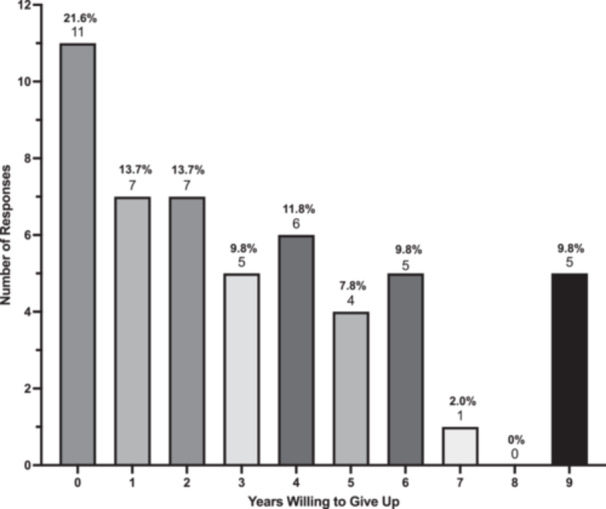
Number of years patients willing to give up to avoid orbital exenteration in time trade‐off task.

**Figure 3:**
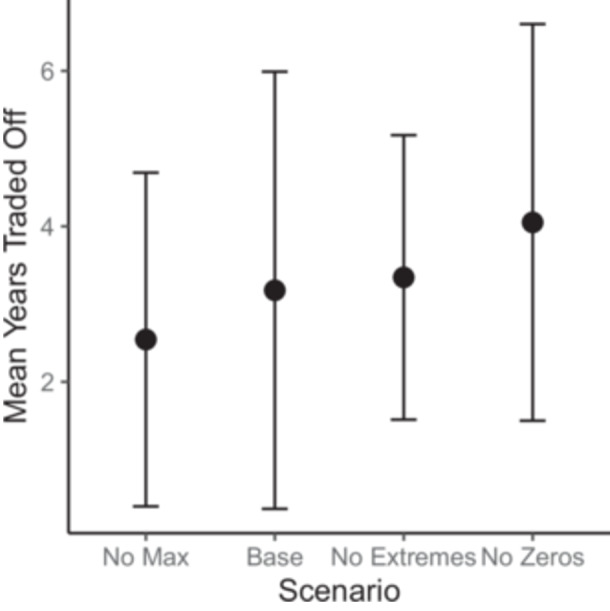
Sensitivity analysis results for time trade‐off.

In the TTO task, patients would give up a mean of 3.2 ± 2.8 years of life to avoid OE. Eleven patients (21.6%) would not give up any years of life, five (9.8%) would give up 9/10 years, and the remaining 36/50 (68.6%) would give up 1 to 7 years (Figure 2). This represents a mean HSUV 0.68 ± 0.28. The sensitivity analysis did not significantly impact the mean HSUV (Figure 3).

**Figure 4:**
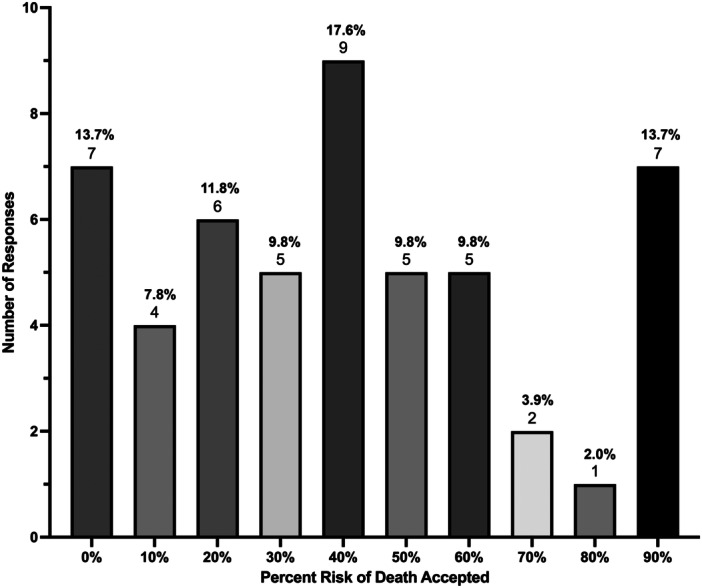
Percent risk of death patients willing to accept to avoid orbital exenteration in the standard gamble task.

In the SG task, patients would accept a mean risk of death of 40.6% ± 28.7% to avoid OE. Seven patients (13.7%) would accept a 90% risk of death, another seven would not accept any (13.7%), and the remaining 37 (72.6%) would accept a risk between 10% and 80% (Figure 4).

**Figure 5:**
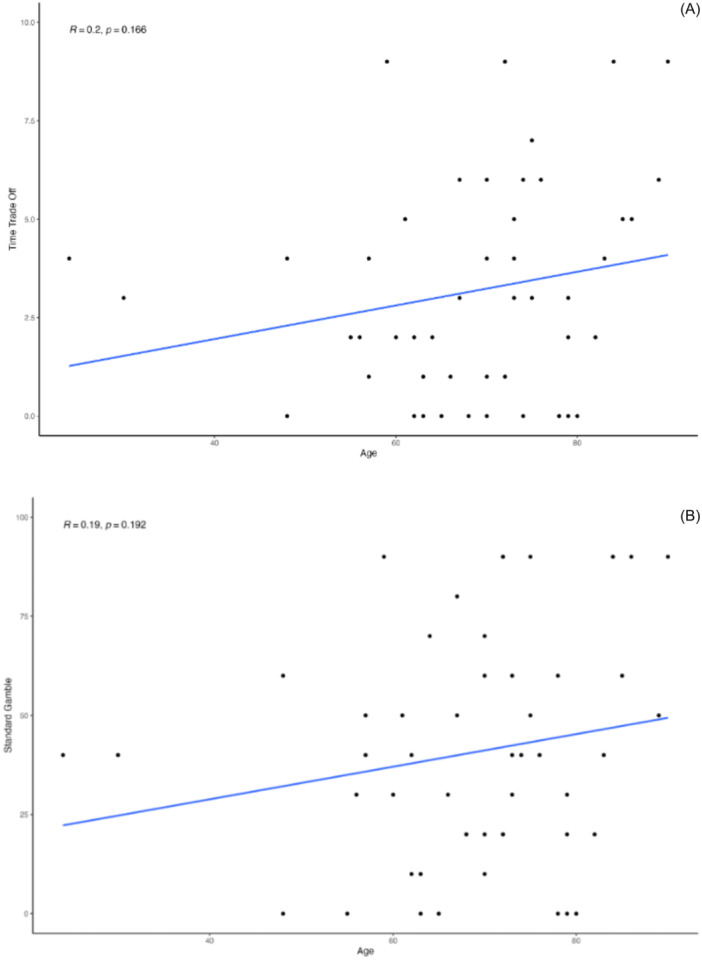
(A, B) Pearson correlation of time trade‐off and standard gamble responses with age.

**Figure 6:**
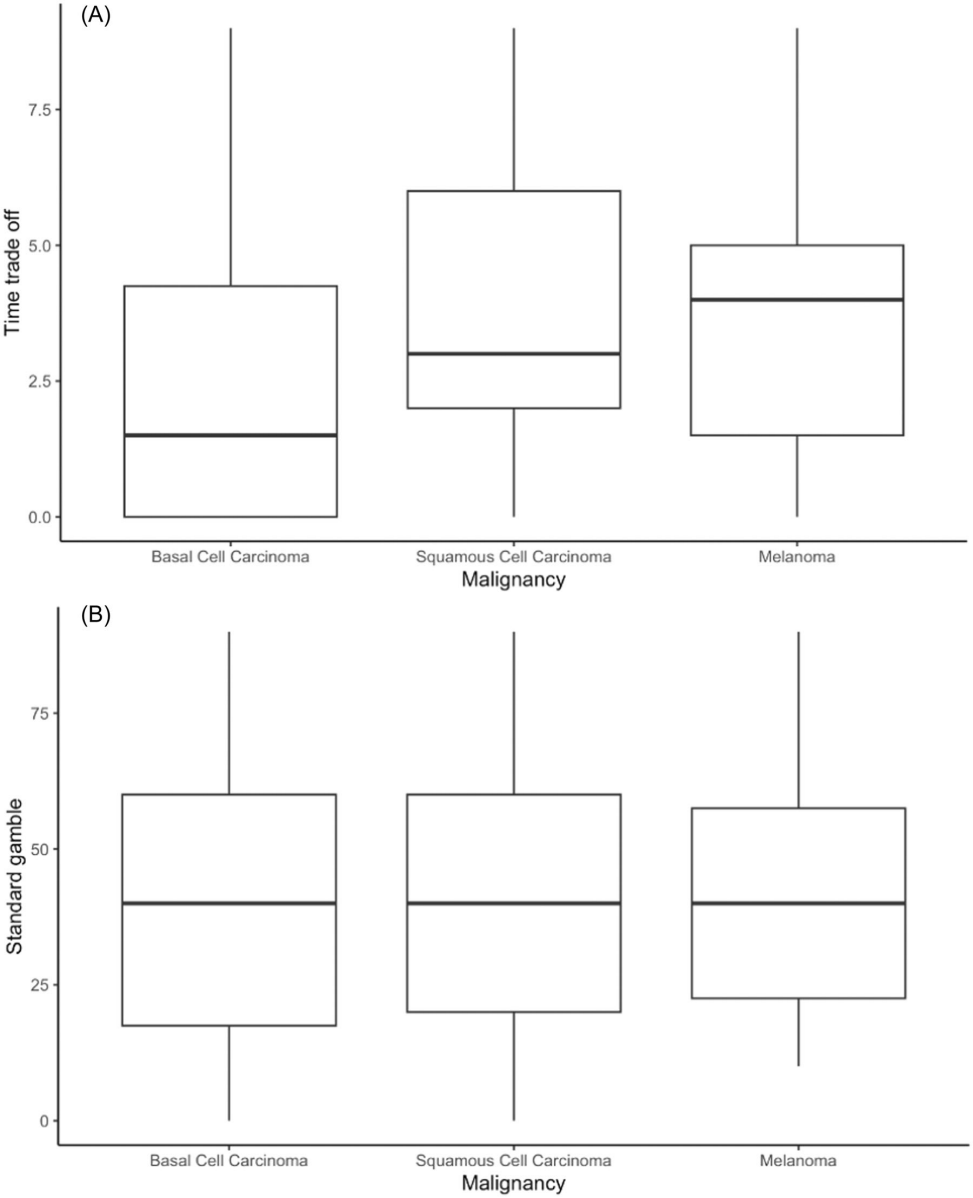
(A, B) Association of type of malignancy with time trade‐off and standard gamble results.

The Pearson correlations showed a weak, but nonsignificant, positive correlation between TTO responses and age (*r*(49) = 0.20, 95% CI = −0.08 to 0.45, *P* = .17) (Figure 5). There was also a weak, but nonsignificant, positive correlation between SG responses and age (*r*(49) = 0.19, 95% CI = −0.09 to 0.44, *P* = .19) (Figure 5). There was no significant correlation between type of malignancy and SG or TTO results based on Kruskal‐Wallis rank sum tests (SG: *H*(2) = 0.20, *P* = .90; TTO: *H*(2) = 3.43, *P* = .18) (Figure 6).

**Figure 7:**
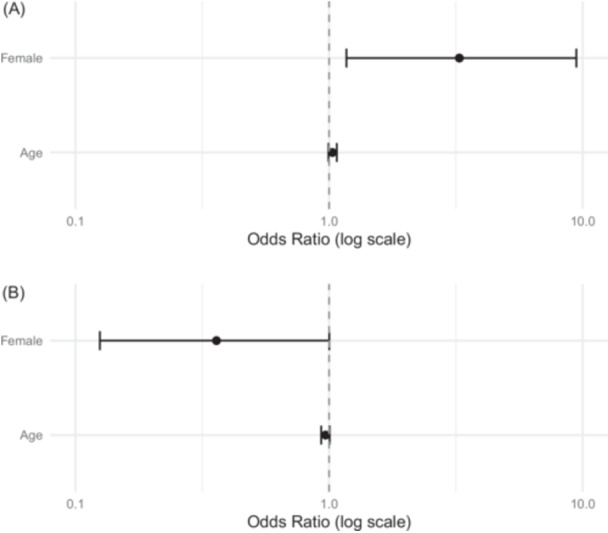
(A, B) Ordered logistic regression results for time trade‐off and standard gamble responses.

The multivariable ordinal logistic regression for TTO found that women were ~3 times more likely to give up an extra year of life when accounting for age (odds ratio [OR] = 3.3, 95% CI = 1.17‐9.45, *P* = .026) (Figure 7). For SG, there were ~64% lower odds of women selecting a lower risk of death to avoid OE, but it was marginally insignificant (OR = 0.36, 95% CI = 0.125‐1.00, *P* = .053) (Figure 7).

### Health Priority Ranking

Most patients ranked quality of life as their top health priority 29/51 (56.9%), followed by length of life 18/51 (35.3%), and then avoiding risks 4/51 (7.8%) (Table 3). The Fisher exact test showed a significant difference in health priority rankings *P* < .0001.

**Table 3:** Health Priority Ranking

Variable	First priority N (%)	Second priority N (%)	Third priority N (%)
Quality of life	29 (56.9)	14 (27.5)	7 (13.7)
Length of life	18 (35.3)	20 (39.2)	14 (27.5)
Avoiding risks	4 (7.8)	17 (33.3)	30 (58.9)

### Qualitative Analysis

Using a base size of 20, we reached a 0% new information threshold at 30^+5^ interviews. The inductive analysis revealed five themes influencing decision‐making: (1) family, (2) healthcare perceptions, (3) age, (4) social consequences, and (5) risk tolerance.

Both the lack and presence of family played a role. Family presence motivated patients to increase time with their family regardless of the self‐impact (eg, I want to see mine [family] grow old like me … I would do anything to have time, as much [time], with them). Patients with dependent family members mentioned accepting reduced health states to continue providing for dependents (eg, … I am the provider for them [family] …). Conversely, patients also wanted to avoid burdening their family with complications and consequences from surgery (eg, I'm ok with things going wrong … but I can't have them [family] doing things for me, that's not me, I don't think that's for me). A lack of family also played a role (eg, I've lived a good life … I'm widowed, my kids don't need me … I don't think I need to go through all that). Finally, patients noted that their family's opinions would impact their decision‐making.

Healthcare perceptions also impacted decision‐making. Prior positive experiences with surgery were noted as a motivator for OE (eg, I had a [procedure] it saved my life, I think anyone would sign up for anything that can do that). Whereas negative experiences decreased willingness for OE (eg, … tough to see [friend] who had [procedure], lots of hospital visits, lots of hospital time, finally got [Medical Assistance in Dying (MAID)]). Similarly, increasing trust and rapport with the healthcare team increased willingness for OE.

Increasing age was a deterrent for OE (eg, … At this [age] I would be happy if I even just lived one more year, if I was younger the story might be different).

There was concern that undergoing OE would impact how others treat them, their self‐perception, and ultimately their confidence. Patients worried that OE may prevent them from engaging in the activities they enjoy (eg, … This may be, I don't know, shallow … I like the way I look, I like to dress up and go out, this would mess with that … I would be worried about what people think … kids would point … I would feel different). Similarly, concerns arose around the impact of securing jobs and making friends.

Finally, risk tolerance impacted decision‐making. Patients noted not wanting this to “linger,” they would rather undergo OE for more “definitive” treatment (eg, that would be so hard, I would think and think and think and worry, I just couldn't).

## Discussion

This is the first study exploring quality versus quantity of life regarding OE. Using TTO and SG procedures, this study found that patients would give up 3.2 ± 2.8 years of life to avoid OE, resulting in an HSUV of 0.68. Patients would also accept a 40.6% ± 28.7% risk of death to avoid OE and trial alternative eye‐sparing strategies. This study provides insight into patient perspectives regarding OE, eye‐sparing strategies, and the factors impacting decision‐making.

Previous studies found HSUVs ranging from 0.54 to 0.95 for laryngeal and oropharyngeal carcinomas.^26^, ^29^, ^33^, ^34^, ^35^, ^43^, ^44^, ^46^, ^47^ The HSUV in this study for OE of 0.68 fits within this range. This suggests the impact of OE on patients' quality of life is in line with total laryngectomy.^29^ However, the HSUV for OE in this study is noticeably lower than the HSUV of 0.95 for minimally invasive transoral robotic surgery and the HSUV of 0.88 for chemoradiotherapy in oropharyngeal carcinoma.^44^ The HSUV of OE was more in line with HSUVs reported from facial disfiguration.^48^, ^49^, ^50^ Severe facial burns were associated with HSUVs of 0.53 to 0.57 and composite facial tissue allotransplantation with HSUVs of 0.56 to 0.67.^48^, ^49^ The HSUVs for post‐rhinectomy without reconstruction were 0.59 to 0.75, postsurgical reconstruction improved this to 0.88 to 0.90, and postsurgical prostheses 0.67 to 0.82.^50^ This demonstrates that the OE HSUV is closer to severe facial disfiguration, including rhinectomy. In this study, surgical reconstruction was mentioned in the TTO and SG tasks for OE, but the HSUV was notably lower than that for reconstruction in rhinectomy, highlighting the persistent deformity associated with OE and its impact on patients. A recent systematic review also noted that heterogeneity between HSUVs can be seen amongst studies examining the same site.^33^ This may be a result of heterogeneity in diagnoses, patients' perceptions, and previous experiences.

This study suggests that patients may be willing to accept reduced oncologic certainty to avoid OE. This impacts discussions regarding eye‐sparing strategies and immunotherapy.^11^ However, patient responses were not uniform and varied overall. Therefore, a single approach cannot be generalized to all patients. This study also sought to understand patient factors influencing decision‐making. Age, complications, and trust in health providers impacted patients and have been reported in previous literature.^34^ However, this study uniquely highlighted the social element of orbital and facial operations. This emphasizes shared decision‐making in head and neck oncology, of which there is a paucity of literature.^51^ These qualitative results may facilitate the development of decision‐making tools to guide patient discussions and facilitate multidisciplinary care.

Although age was a recurring theme in the qualitative results, it was not significantly associated with patient responses. The multivariable analysis did show that patients who identified as women were willing to give up more years of life or accept an increased risk of death to avoid OE. This theme is present in previous literature where younger and female respondents are noted to perceive a higher impact on their self‐perception.^52^ Central facial lesions also had a greater impact.^52^ Despite these findings, clinical situations should be approached individually to avoid stereotyping.

The HSUVs provide quantitative measures of the impact of OE. Eye‐sparing strategies center on immunotherapy, which can be costly. HSUVs can be converted to quality‐adjusted life‐years (QALY) by multiplying the reduction in HSUV (1 − 0.68 = 0.32) by the number of years of life remaining. The mean age in this study was 69 years, and the average life expectancy in Canada is 81, resulting in a mean remaining life expectancy of 12 years (81 − 69 = 12).^53^ The total gain in QALY would be 3.8 (0.32 × 12 = 3.8). Cost‐effectiveness threshold per QALY is $100,000.^54^ Therefore, $100,000 × 3.8 provides a cost‐effectiveness threshold of $380,000. Although these discussions are more complex given the risks of immunotherapy, treatment failure, and systemic toxicity, this provides a framework to further engage in this discussion.

It is important to note that this and previous HSUV studies simplify the decision‐making to binary options: OE or no OE. Given the complex outcomes of treatments, multiple health states may impact decision‐making. This was recently assessed for laryngeal cancer, by describing health states post‐chemoradiotherapy and total laryngectomy with optimal outcomes or complications.^29^ When describing chemoradiotherapy or total laryngectomy with optimal outcomes, HSUVs were similar to this study: 0.64 and 0.57, respectively.^29^ When described with complications, the HSUVs decreased to 0.31 and 0.33, respectively.^29^ Highlighting the impact of complications on patients' quality of life. This study explained potential complications of OE to try to mimic how patients may approach the situation when experiencing it firsthand. However, optimal health states were not separated from those with complications. This is an important next step to build off the current work. Specifically, including suboptimal health states in the context of complications post‐OE and systemic toxicities from immunotherapy.

This study is limited by its hypothetical nature. The procedure and pathology of OE were described in depth but cannot mimic a patient's first‐hand decision‐making. Systemic toxicities with immunotherapy were mentioned, but not specifically described. Patient perceptions may be biased as all were treated by a single surgeon, limiting generalizability. Patient trust, healthcare perceptions, and ultimately decision‐making are influenced by their experiences, which are impacted by their shared experience with the study's senior author. This study excluded patients who had previously undergone OE; this allowed for a more homogenous sample and helped mimic the decision a patient may have to make firsthand. However, it excludes the perspectives of OE patients. We aim to explore this further as well as decisional regret amongst OE patients. The sample size of 51 met our power requirements, but a larger sample may provide more robust results. Finally, the tasks in this study are complex, but utilizing videoconferencing allowed patients to grasp the nature of the study. Nevertheless, this study provides a unique framework to assess OE and eye‐sparing strategies from patients' perspectives.

## Conclusion

When patients are presented with less certainty of oncologic control, some are willing to accept this trade‐off to avoid OE. On average, patients accepted a 40% risk of death or a reduced lifespan of 3.2 years to avoid OE. This highlights the importance of eye‐sparing strategies, the use of immunotherapy, and shared decision‐making regarding OE as patient priorities may not be solely related to survival.

## Author Contributions

**Kalpesh Hathi**, Study design, interviewing patients, collecting data, analyzing results, interpreting results, drafting manuscript, revising manuscript. **Colin MacKay**, Analyzing data, interpreting data, drafting manuscript, revising manuscript. **Robyn Macfarlane**, Interpreting data, revising manuscript. **S. Mark Taylor**, Study design, interpreting data, revising manuscript, supervision.

## Disclosures

### Competing interests

The authors declare no conflicts of interest upcoming or existing in the previous 24 months that pertain to this article.

### Funding source

None.

## Supporting information

Supporting Information.
