# Supplementary material for: Patient Perspectives of Quality Compared to Quantity of Life Regarding Orbital Exenteration
Source: Otolaryngol Head Neck Surg. 2025 Jul 29;173(5):1170–81. doi: 10.1002/ohn.1364 (PMC12574635; doi:10.1002/ohn.1364)
Supplement: Supplementary file 1 — Supporting Information. [file OHN-173-1170-s001.docx]

**Appendix A: Questionnaire/Interview Script**

Thank you for agreeing to participate in our research study. Over the next 15-20 minutes we will go through a series of questions and tasks. Some may seem like odd questions, but we are looking for your natural reaction to questions and scenarios to give us better information on the care we provide patients and their reactions to it.

At any time if you have any questions, are confused about what we are asking you, or would like to stop, please just let us know.

This first section tells us a bit more about you:

**Demographics**:

What is your self-identified gender?

1. Male
2. Female
3. Other

What is your highest level of education?

1. Did not complete high school
2. High school graduate
3. GED
4. Community college degree
5. Bachelor’s degree
6. Trade’s education
7. Master’s degree or higher

What is your average household income per year?

1. <25,000$
2. 25,000-49,999$
3. 50,000-99,999 $
4. 100,000-149,999$
5. 150,000-249,999$
6. 250,000-500,000$
7. >500,000$

This next section asks you how you would react when given two scenarios.

This study largely focuses on people’s reactions to a surgery called orbital exenteration. This surgery is performed for skin cancer that spreads to the eye. The surgery involves removing your eye, and surrounding structures, including your eyelids. Often skin from other parts of your body such as your arm or leg is taken to cover the defect from removing your eye.

This results in cosmetic changes to your face as well as loss of vision in one eye. We are interested in how long people would give up living to avoid this surgery.

It is also important to note that this is a large surgery, which results in patients being in hospital for 1-4 weeks on average. All surgeries have risks, this one includes infections, bleeding, need for further surgeries, injuries to surrounding structures such as other parts of your face, numbness around the surgical site, decreased movement of your face, injury to the brain, death, risk of not getting all the cancer out, heart attack, blood clots in lung, and chronic pain.

To get a better idea of what life would be like after this surgery we have some pictures of what people may look like after the surgery and after they have healed. As you will see there are options such as prostheses to minimize the cosmetic effects.

**Time-Trade Off Task:**

In this next section we would like to understand how you feel about the idea of undergoing this procedure and if you would give up part of your life span to keep your eye and avoid this procedure. For this section consider “good health” to be your health right now.

Question 1

Would you rather:

1. Live a **guaranteed 10 full years in your current health with no eye** after having this procedure and **accepting its associated risks**.

OR

1. Live a **guaranteed full 9 years without having this procedure and keeping your eye.**

Question 2

Would you rather:

1. Live a **guaranteed 10 full years in your current health with no eye** after having this procedure and **accepting its associated risks**.

OR

1. Live **a guaranteed full 8 years without having this procedure and keeping your eye.**

Question 3

Would you rather:

1. Live a **guaranteed 10 full years in your current health with no eye** after having this procedure and **accepting its associated risks**.

OR

1. Live a **guaranteed full 7 years without having this procedure and keeping your eye.**

Question 4

Would you rather:

1. Live a **guaranteed 10 full years in your current health with no eye** after having this procedure and **accepting its associated risks**.

OR

1. Live a **guaranteed full 6 years without having this procedure and keeping your eye.**

Question 5

Would you rather:

1. Live a **guaranteed 10 full years in your current health with no eye** after having this procedure and **accepting its associated risks**.

OR

1. Live a **guaranteed full 5 years without having this procedure and keeping your eye.**

Question 6

Would you rather:

1. Live a **guaranteed 10 full years in your current health with no eye** after having this procedure and **accepting its associated risks**.

OR

1. Live a **guaranteed full 4 years without having this procedure and keeping your eye.**

Question 7

Would you rather:

1. Live a **guaranteed 10 full years in your current health with no eye** after having this procedure and **accepting its associated risks**.

OR

1. Live a **guaranteed full 3 years without having this procedure and keeping your eye.**

Question 8

Would you rather:

1. Live a **guaranteed 10 full years in your current health with no eye** after having this procedure and **accepting its associated risks**.

OR

1. Live a **guaranteed full 2 years without having this procedure and keeping your eye.**

Question 9

Would you rather:

1. Live a **guaranteed 10 full years in your current health with no eye** after having this procedure and **accepting its associated risks**.

OR

1. Live a **guaranteed full 1 year without having this procedure and keeping your eye.**

**Standard Gamble Task:**

This next section is similar to the previous where you will be given two options and will have to decide between the two. We would like to know if you would prefer to live a given period of time with no eye after having the procedure or take a pill that would let you live at least 10 years but brings with it a certain risk of death

Question 1

Would you rather:

1. Live a **guaranteed 10 full years in your current health with no eye** after having this procedure and **accepting its associated risks**.

OR

1. Take a pill that has **90%** risk of death but a **10%** chance of living 10 years in full health.

Question 2

Would you rather:

1. Live a **guaranteed 10 full years in your current health with no eye** after having this procedure and **accepting its associated risks**.

OR

1. Take a pill that has **80%** risk of death but a **20%** chance of living 10 years in full health.

Question 3

Would you rather:

1. Live a **guaranteed 10 full years in your current health with no eye** after having this procedure and **accepting its associated risks**.

OR

1. Take a pill that has **70%** risk of death but a **30%** chance of living 10 years in full health.

Question 4

Would you rather:

1. Live a **guaranteed 10 full years in your current health with no eye** after having this procedure and **accepting its associated risks**.

OR

1. Take a pill that has **60%** risk of death but a **40%** chance of living 10 years in full health.

Question 5

Would you rather:

1. Live a **guaranteed 10 full years in your current health with no eye** after having this procedure and **accepting its associated risks**.

OR

1. Take a pill that has **50%** risk of death but a **50%** chance of living 10 years in full health.

Question 6

Would you rather:

1. Live a **guaranteed 10 full years in your current health with no eye** after having this procedure and **accepting its associated risks**.

OR

1. Take a pill that has **40%** risk of death but a **60%** chance of living 10 years in full health.

Question 7

Would you rather:

1. Live a **guaranteed 10 full years in your current health with no eye** after having this procedure and **accepting its associated risks**.

OR

1. Take a pill that has **30%** risk of death but a **70%** chance of living 10 years in full health.

Question 8

Would you rather:

1. Live a **guaranteed 10 full years in your current health with no eye** after having this procedure and **accepting its associated risks**.

OR

1. Take a pill that has **20%** risk of death but a **80%** chance of living 10 years in full health.

Question 9

Would you rather:

1. Live a **guaranteed 10 full years in your current health with no eye** after having this procedure and **accepting its associated risks**.

OR

1. Take a pill that has **10%** risk of death but a **90%** chance of living 10 years in full health.

**Health Priority Ranking:**

When choosing between the options in the previous two tasks, out of these three options can you rank them on importance to you from 1-3 (1 being the most important)

- Quality of life: Keeping your eye and avoiding the surgery
- Length of life: Living for as long as possible
- Avoiding risks

Are there any other factors that came to your mind? If so, what?
